# Urodynamic prognostic factors for large post-void residual urine volume after intravesical injection of onabotulinumtoxinA for overactive bladder

**DOI:** 10.1038/srep43753

**Published:** 2017-03-06

**Authors:** Sheng-Mou Hsiao, Ho-Hsiung Lin, Hann-Chorng Kuo

**Affiliations:** 1 Department of Obstetrics and Gynecology, Far Eastern Memorial Hospital, Banqiao, New Taipei, Taiwan; 2 Graduate School of Biotechnology and Bioengineering, Yuan Ze University, Taoyuan, Taiwan; 3 Department of Obstetrics and Gynecology, National Taiwan University College of Medicine and National Taiwan University Hospital, Taipei, Taiwan; 4 Department of Urology, Buddhist Tzu Chi General Hospital and Tzu Chi University, Hualien, Taiwan

## Abstract

The aim of this study was to identify factors predicting large post-void residual (PVR) (defined as ≥200 mL), an important unsolved problem, after an intravesical injection of onabotulinumtoxinA in patients with overactive bladder syndrome. The data showed that 133 of 290 patients had a large PVR after treatment. Multivariate analysis found that the baseline 3-day daytime frequency episodes and voiding efficiency were independent predictors for postoperative large PVR. A receiver operating characteristic (ROC) curve analysis showed the following optimum cut-off values: (1) 3-day daytime frequency episodes = 25, which has a ROC area of 0.72; and (2) voiding efficiency = 89%, which has a ROC area being 0.66. The predicted logit transformation of probability of large PVR, logit(p), for a given 3-day daytime frequency episodes (*a*) and voiding efficiency (*b*%) can be denoted by logit(p) = −5.18 + 0.07* *×* a* + 0.04* *×* b*, with a cutoff value of logit(p) = 0.34 and a ROC area of 0.79. The median value of the persistent large PVR interval was 5 months. In conclusion, low 3-day daytime frequency episodes (<25) and low voiding efficiency (<89%) are associated with large PVR. Besides, logit(p) <0.34 can be used to predict large PVR for its higher ROC area.

Overactive bladder syndrome (OAB) is characterized by urgency with or without urge incontinence and is usually associated with frequency and nocturia^1^. Antimuscarinic agents are the first-line treatment, with a success rate greater than 70%^2^. Both the presence of urothelial dysfunction and abnormalities of sensory receptor expression or transmitter release in the suburothelial nerves may contribute to OAB, which is refractory to antimuscarinics^3^. Thus, intravesical treatment to inhibit abnormal receptor expression or transmitter release in the suburothelial space has therapeutic effects in OAB patients^4^.

The intravesical injection of onabotulinum toxin type A has emerged as the treatment of choice in cases with OAB refractory to antimuscarinic therapy. The intravesical injection of onabotulinum toxin type A has both motor and sensory effects^5^. Despite the acceptable therapeutic effects obtained following the intravesical injection of onabotulinum toxin type A in OAB cases, the resulting detrusor contractility impairment and large post-void residual (PVR) remains an important unsolved problem for patients^6^,^7^,^8^,^9^.

Previous reports have demonstrated that large PVR develops significantly more often in men and in patients >61 years old. Large PVR is also observed in patients with a baseline maximum flow rate (Qmax) ≤15 ml/sec, PVR ≥ 100 ml, and voiding efficiency <90% after the intravesical injection of onabotulinum toxin type A^10^. Additionally, Liao *et al*. reported an increased risk of large PVR in frail elderly patients^11^. Wang *et al*. reported that diabetic patients had a significantly greater incidence of large PVR after treatment^12^. Furthermore, Kuo *et al*. found that the incidence of post-treatment large PVR was associated with medical comorbidities^13^. The study by Osborn *et al*. reported that the preoperative PVR and bladder capacity were associated with postoperative urine retention in a multivariate logistic regression^14^. However, age and diabetes were not associated with postoperative urine retention^14^. These conflicting results led us to identify the independent factors that can predict large PVR after intravesical onabotulinum toxin type A injection for patients with OAB. In addition, the secondary objective was to estimate the recovery time after the presence of postoperative large PVR and identify factors responsible for recovery.

## Results

There were 290 patients who underwent intravesical onabotulinum toxin type A injection for OAB, and 133 patients (45.9%, male, n = 65 vs. female, n = 68, P = 0.50) who experienced large PVR (i.e., >200 mL) during the 6-month follow-up period.

Univariate analysis revealed the following baseline characteristics that were predictors for post-treatment large PVR: age, daytime frequency episodes, PVR, voiding efficiency and cystometric bladder capacity (Table 1). However, only daytime frequency episodes and voiding efficiency were independent predictors for post-treatment large PVR by backward stepwise multivariate logistic regression analysis (Table 1).

**Figure 1:**
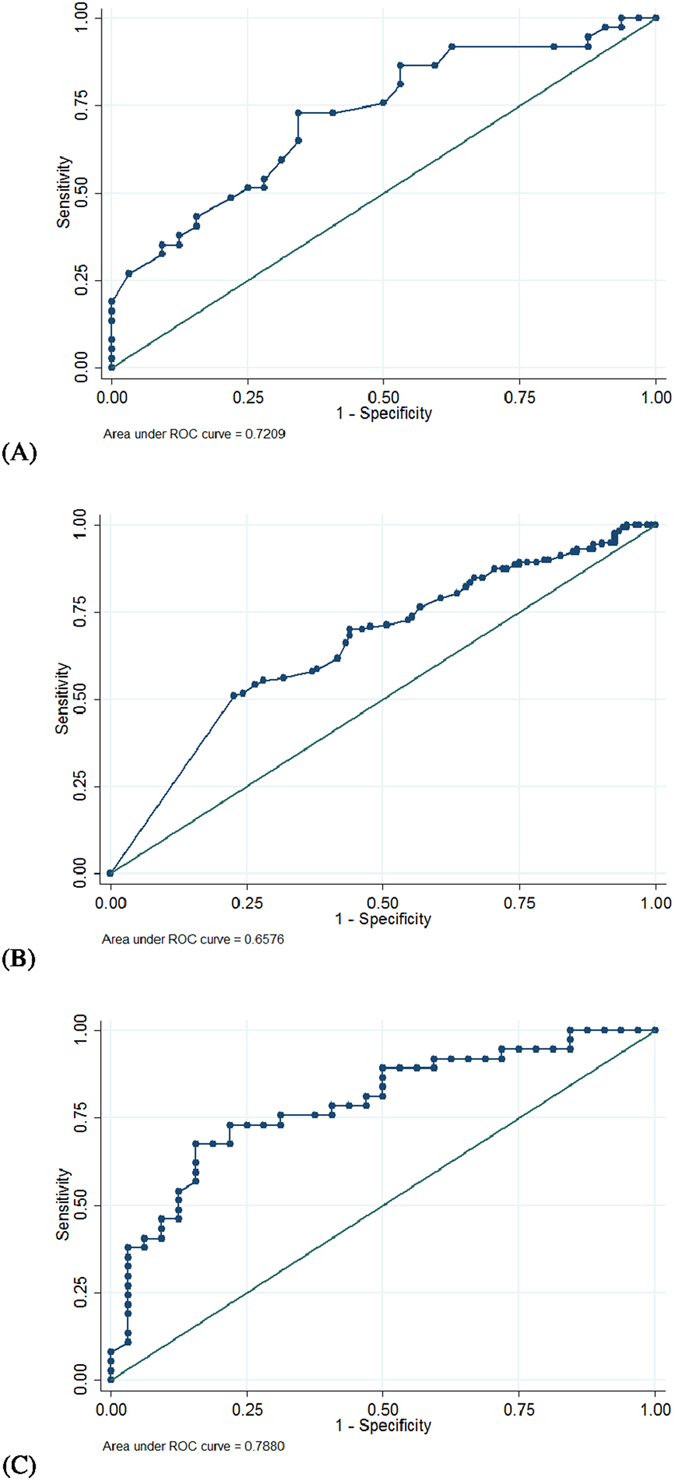
The receiver operating characteristic (ROC) curves of using (**A**) 3-day daytime frequency episodes, (**B**) voiding efficiency (%) and (**C**) logit(p) to predict postoperative large post-void residual.

The following optimum cut-off values were determined using ROC analysis: (1) baseline 3-day daytime frequency episodes = 25, which has an area under the ROC curve of 0.72 (95% confidence interval [CI] = 0.60 to 0.84; sensitivity = 73.0%, specificity = 65.6%, Fig. 1A); and (2) baseline voiding efficiency = 89%, which has an area under the ROC curve of 0.66 (95% CI = 0.60 to 0.72; sensitivity = 70.1%, specificity = 56.1%, Fig. 1B).

By multivariate logistic regression analysis, baseline 3-day daytime frequency episodes (coefficient = 0.07, *P* = 0.005) and baseline voiding efficiency (coefficient = 0.04, *P* = 0.03) were determined to be the significant independent factors to predict the probability of the presence of large PVR with a constant of −5.18 (95% confidence interval [CI] = −8.66 to −1.69, *P* = 0.004). Thus, the predicted logit(p) for a given baseline 3-day daytime frequency episodes (a) and baseline voiding efficiency (*b*%) can be denoted by logit(p) = −5.18 + 0.07* *×* a* + 0.04* *×* b*. The mean and standard deviation of logit(p) was 0.14 and 1.31, respectively. Based on the ROC analysis, the optimum cut-off values of logit(p) = 0.34 were determined with the area under the ROC curve being 0.79 (95% confidence interval [CI] = 0.68 to 0.90; sensitivity = 67.6%, specificity = 84.4%, Fig. 1C).

**Figure 2:**
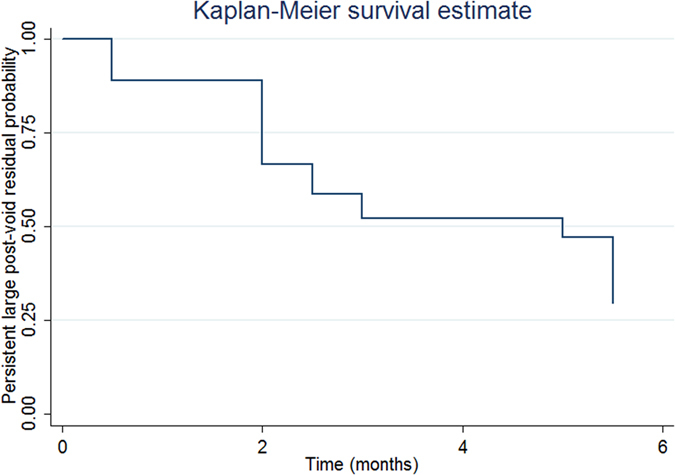
Persistent large post-void residual probability in all patients with overactive bladder syndrome who underwent intravesical onabotulinum toxin type A injection.

The median value of the persistent large PVR interval was 5 months (95% CI = 2.5 to 5.5 months, Fig. 2). The baseline variables including age, gender, comorbidities, overactive bladder symptoms score (OABSS), bladder diary and video-urodynamic variables were not significant factors associated with predicting the persistent large PVR interval.

**Figure 3:**
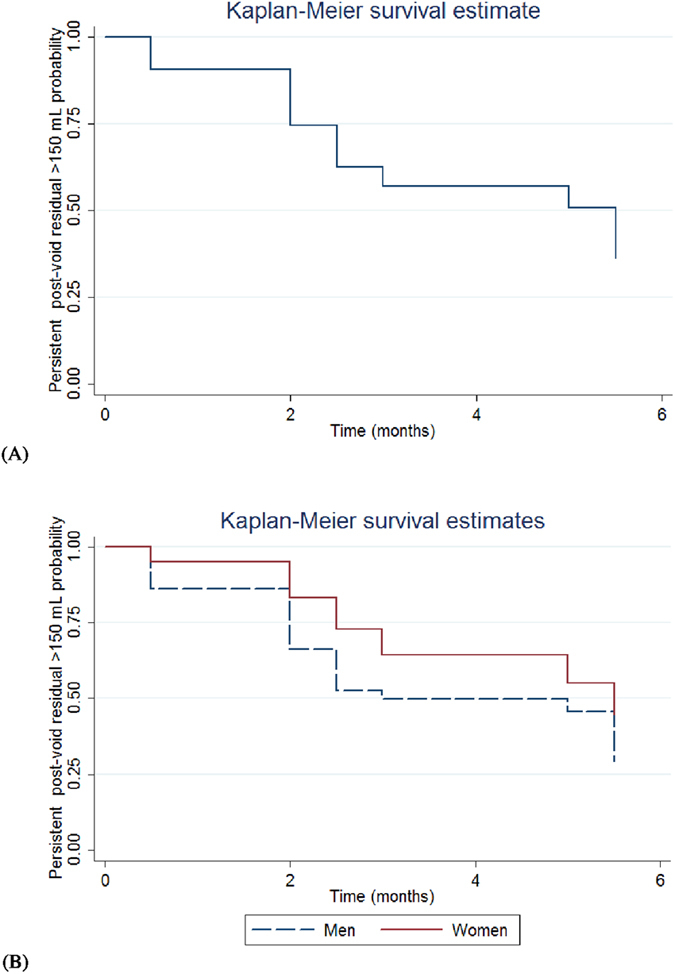
Persistent post-void residual >150 mL probability in (**A**) all patients and (**B**) both genders with overactive bladder syndrome underwent intravesical onabotulinum toxin type A injection.

If we defined large PVR as >150 mL^11^, instead of >200 mL, 163 patients (56.2%) had PVR > 150 mL during the 6-month follow-up period. The baseline 3-day daytime frequency episodes and baseline voiding efficiency were the only significant independent predictors for post-treatment PVR > 150 mL, with similar odds ratios of 0.93 (95% CI = 0.89 to 0.98) and 0.93 (95% CI = 0.88 to 0.97), respectively. The ROC areas were 0.71 and 0.66, respectively. The median persistent PVR > 150 mL interval is 5.5 months (95% CI = 3 to 5.5 months, Fig. 3A). The data show that the male patients had a shorter persistent PVR > 150 mL interval than the female patients (median: male, 3 months, 95% CI = 2.5 to 5.5 months vs. female, 5.5 months, 95% CI = 5 to - months, log-rank test, P = 0.04, Fig. 3B).

## Discussion

We successfully identified low voiding efficiency (<89%) as an independent factor for predicting post-treatment large PVR. However, variables such as age, comorbidities (including diabetes mellitus), baseline Qmax, PVR, Pdet.Qmax or bladder contractility index were not predictive factors. Previous studies have shown that age, comorbidities, baseline Qmax, PVR, Pdet.Qmax and bladder contractility index are associated with large PVR after intravesical onabotulinum toxin type A injection^10^,^11^,^12^,^13^,^14^. Comorbidities such as diabetes mellitus and Pdet.Qmax were not statistically significant in our univariate analysis (Table 1). Although age, baseline Qmax, PVR and bladder contractility index were statistically significant in our univariate analysis (Table 1), these variables failed to remain significant in multivariate analysis (Table 1). The finding of low voiding efficiency as a predictor is similar to the data reported by Jiang *et al*.^10^ and Hsiao *et al*.^15^.

Osborn *et al*. reported that preoperative PVR and bladder capacity were associated with postoperative urine retention in a multivariate logistic regression^14^. It is notable that if we excluded voiding efficiency as a variable in our multivariate backward stepwise logistic regression model, then baseline PVR becomes an independent predictor (odds ratio = 1.01, P = 0.04). This finding would be consistent with the data reported by Osborn *et al*.^14^. However, cystometric bladder capacity remains insignificant as a predictor. PVR and bladder capacity are components of voiding efficiency. Thus, voiding efficiency should be a better predictor than PVR and bladder capacity^14^.

We also identified a novel predictor (daytime frequency episodes) of post-treatment large PVR (Table 1). Low daytime frequency episodes (<25 episodes during 3 days) can also predict a high incidence of large PVR (Table 1, Fig. 1A). We excluded patients with detrusor underactivity for intravesical onabotulinum toxin type A injection; thus our OAB patients in this study should have intact bladder contractility^16^. From our finding (Table 1, Fig. 1A), the bladder contractility of OAB patients with high daytime frequency episodes would be less affected by onabotulinum toxin type A injection.

Previous studies reported that the following characteristics were associated with large PVR after treatment: male patients, >61 years old, baseline Qmax ≤15 mL/sec, PVR ≥ 100 mL, voiding efficiency <90%, comorbidities, and diabetes^10^,^11^,^12^,^13^. However, only comorbidities were identified by multivariate logistic regression analysis^13^, the other studies did not perform multivariate analysis^10^,^11^,^12^. Thus, our predictors for post-treatment large PVR should be more valuable and reliable.

In a previous study^13^, comorbidities (including diabetes mellitus, chronic kidney disease, chronic obstructive pulmonary disease and congestive heart failure in previous study) predicted large PVR (odds ratio = 2.2, P = 0.01)^13^. However, all comorbidities are not a predictor in the current study (odds ratio = 1.20, P = 0.45, Table 1). If we use 4 comorbidities (including variables of diabetes mellitus, chronic kidney disease, chronic obstructive pulmonary disease and congestive heart failure as previous study^13^), instead of all cormorbidities (Table 1), the variable remains insignificant as a predictor of large PVR (odds ratio = 1.23, P = 0.38). Thus, the impact of comorbidities on the post-treatment PVR requires further clarification.

We also found that the median bladder recovery time for patients experiencing large PVR is 5 months (Fig. 2), despite the intravesical onabotulinum toxin type A injection may remain therapeutically effective in patients for up to 1 year^13^. Besides, from the finding of Fig. 3B, male patients had a faster recovery from large PVR than female patients. These data should be valuable for patient consultations.

A limitation of this study is the retrospective design. However, the large sample size and multivariate backward stepwise logistic regression analysis performed in this study should make our analysis reliable and valuable. In addition, because the ROC areas of daytime frequency episodes and voiding efficiency were low, the proposed cut-off values might be less useful. However, logit(p) had a higher ROC area, thus the proposed cut-off value (0.34) of logit(p) can provide a valuable reference for physicians to select appropriate OAB patients for intravesical onabotulinum toxin type A injection.

In conclusion, low 3-day daytime frequency episodes (<25) and low voiding efficiency (<89%) are associated with large PVR. Besides, logit(p) <0.34 can be used to predict large PVR for its higher ROC area. The median recovery time for large PVR was 5 months. Our findings can be used as an initial guide for intravesical onabotulinum toxin type A injection in patients with refractory OAB.

## Methods

This study was a retrospective investigation of 290 OAB patients who were treated with intravesical injection of 100 U of onabotulinum toxin type A (Allergan, Irvine, CA, USA) from 2005 to 2014. All patients received their first onabotulinum toxin type A injection.

The indications of intravesical onabotulinum toxin type A injection were OAB patients who were refractory to antimuscarinic treatment. The duration of previous antimuscarinic treatment was at least 3 months. All patients had been treated with at least two different antimuscarinic agents and were still bothered by severe urgency or urgency incontinence of at least one episode per day. All patients were free of urinary tract infections, intrinsic sphincter deficiency, and neurogenic bladder at the time of study enrolment.

The Research Ethics Committee of Hualien Tzu Chi Hospital approved the study. The corresponding author confirmed that all methods were performed in accordance with relevant guidelines and regulations. This study is a post hoc analysis of a previous study^10^. All patients were informed about the possible adverse events after onabotulinum toxin type A injection, and written informed consent was obtained from all patients before treatment. All patients received intravesical suburethelial injections totalling 100 U of onabotulinum toxin type A in the bladder body and excluding the trigone.

The video-urodynamic study was routinely performed for the diagnosis of detrusor over activity, bladder outlet obstruction, and intrinsic sphincter deficiency using Life-Tech urodynamics equipment (Stafford, Texas, USA). Women with bladder outlet obstruction and detrusor underactivity were also excluded from this study. A bladder outlet obstruction was defined as the radiologic evidence of bladder outlet narrowing, a voiding detrusor pressure greater than 35 cmH2O and a Qmax less than 15 mL/s or a voiding detrusor pressure greater than 40 cmH2O^17^. If patients did not have a voiding detrusor contractility of more than 10 cmH2O and needed to void by abdominal straining or were unable to void, then detrusor underactivity was diagnosed^18^. The urodynamic parameters of the first sensation of filling, full sensation, cystometric bladder capacity, bladder compliance, Qmax, PVR, voiding detrusor pressure at Qmax (Pdet.Qmax), bladder contractility index (defined as Pdet.Qmax +5 × Qmax)^19^, and voiding efficiency (defined as voided volume/ bladder capacity ×100%)^19^ were measured and recorded in detail.

A three-day voiding diary^20^, uroflowmetry for Qmax, voided volume and PVR was performed at each visit. PVR was measured by transabdominal ultrasound at each visit to the outpatient clinic. The bladder capacity was derived from the sum of the voided volume and PVR.

The urgency severity scale (USS) was measured using a modified version of the validated Indevus Urgency Severity Scale. The USS rates urgency severity as 0, 1, 2, or 3 and is defined as none, mild, moderate, and severe urgency, respectively^21^. We defined urgency incontinence as an urgency incontinence score of 4. The overactive bladder symptom score (OABSS)^22^ was also measured at each visit. OAB-wet was diagnosed by the presence of at least one episode of urgency incontinence in the 3-day voiding diary. The remaining patients were considered OAB-dry.

The therapeutic efficacy was graded using the Global Response Assessment (GRA) and categorized as −3, −2, −1, 0, 1, 2, and 3, indicating markedly worse, moderately worse, mildly worse, no change, mildly improved, moderately improved, and markedly improved bladder symptoms, respectively. The patients were closely monitored by personal interview and reported their subjective perception of the bladder condition at 2 weeks, 1, 3, and 6 months after the onabotulinum toxin type A injection. These results were combined with the improvement of OAB symptoms and any adverse effects after onabotulinum toxin type A treatment. A GRA score ≥2 at 3 months after onabotulinum toxin type A injection was defined as a successful treatment^23^.

STATA software (Version 11.0; Stata Corp, College Station, TX, USA) was used for statistical analyses. The variables in the univariate regression analysis included age, gender, OAB-wet or OAB-dry, comorbidities, OABSS, USS scores, 3-day voiding diary and urodynamic variables. Multivariate backward stepwise logistic regression analysis was performed using the variables with P < 0.25 from univariate analysis^24^. A *P* value of less than 0.05 was considered statistically significant. Receiver operating characteristic (ROC) curve analysis was performed to identify the optimum cut-off value for predicting post-treatment large PVR (defined as ≥200 mL). The optimal cut-off value was determined by the point on the ROC curve closest to the upper left-hand corner. In addition, multivariate logistic regression analysis including all significant factors was performed to predict the probability of large PVR (=p) and obtain logit transformation logit(p)^25^,^26^. With all patients’ values derived from the equation of logit(p), ROC curve analysis was performed to identify optimum cutoff value.

The persistent large PVR interval was measured from the date of first documented large PVR during the follow-up visits to the date of documented absence of large PVR and afterwards or last follow-up. The survival curve of the persistent large PVR interval was estimated using the Kaplan-Meier method. The P values of between-group differences were calculated from the log-rank test^27^. A Cox proportional-hazards model was used to assess the factors affecting the persistent large PVR interval.

## Additional Information

**How to cite this article**: Hsiao, S.-M. *et al*. Urodynamic prognostic factors for large post-void residual urine volume after intravesical injection of onabotulinumtoxinA for overactive bladder. *Sci. Rep.* **7**, 43753; doi: 10.1038/srep43753 (2017).

**Publisher's note:** Springer Nature remains neutral with regard to jurisdictional claims in published maps and institutional affiliations.

## Figures and Tables

**Table 1:** Baseline characteristics and logistic analysis for predicting large post-void residual after intravesical onabotulinum toxin type A injection for overactive bladder (n = 290).

Variable	Values	Univariate		†Multivariate	
		Odds ratio	P	Odds ratio	P
Age (years)	68.0 ± 14.8	1.02 (1.01–1.04)	0.01	—	—
Men	148 (51)	0.85 (0.54–1.35)	0.50		
Women	142 (49)	—	—	—	—
OAB wet	248 (86)	1.29 (0.66–2.51)	0.45		
OAB dry	42 (14)	—	—	—	—
CKD	30 (10)	1.20 (0.56–2.56)	0.63	—	—
COPD	10 (3)	1.19 (0.34–4.19)	0.79	—	—
CAD	32 (11)	0.79 (0.37–1.66)	0.53	—	—
Diabetes mellitus	91 (31)	1.08 (0.66–1.78)	0.75	—	—
CHF	42 (14)	1.36 (0.71–2.61)	0.36	—	—
CVA	52 (18)	1.11 (0.61–2.03)	0.72	—	—
Parkinsonism	10 (3)	1.19 (0.34–4.19)	0.79	—	—
Dementia	28 (10)	1.41 (0.64–3.08)	0.39	—	—
All comorbidities	150 (52)	1.20 (0.75–1.90)	0.45		
OABSS	11.8 ± 2.4	0.93 (0.76–1.13)	0.44	—	—
USS	3.8 ± 0.5	1.74 (0.67–4.49)	0.26	—	—
PPBC	4.6 ± 1.7	0.96 (0.72–1.27)	0.77	—	—
Daytime frequency episodes (72 h)	29.0 ± 13.3	0.93 (0.89–0.98)	0.003	0.93 (0.89–0.98)	0.01
Nocturia episodes (72 h)	9.9 ± 4.9	0.94 (0.85–1.04)	0.25	—	—
Urgency episodes (72 h)	29.1 ± 15.8	0.97 (0.94–1.00)	0.07	—	—
Urgency incontinence episodes (72 h)	8.2 ± 11.0	0.97 (0.92–1.02)	0.17	—	—
Maximum flow rate (mL/s)	13.1 ± 8.0	0.98 (0.95–1.01)	0.18	—	—
Voided volume (mL)	207 ± 114	1.000 (0.998–1.002)	0.74	—	—
Post-void residual (mL)	41 ± 72	1.009 (1.004–1.014)	<0.001	—	—
Bladder capacity (mL)	248 ± 124	1.002 (1.000–1.004)	0.053	—	—
Voiding efficiency (%)	84.8 ± 19.9	0.97 (0.96–0.99)	<0.001	0.96 (0.93–1.00)	0.03
First sensation of filling (mL)	114 ± 64	1.01 (1.00–1.01)	0.06	—	—
Cystometric bladder capacity (mL)	240 ± 124	1.003 (1.001–1.005)	0.003	—	—
Detrusor overactivity	282 (97)	0.84 (0.21–3.44)	0.81	—	—
Pdet.Qmax (cmH2O)	27.1 ± 15.2	1.00 (0.99–1.02)	0.68	—	—
Bladder contractility index	92.2 ± 42.9	1.00 (0.99–1.00)	0.22	—	—

Values were expressed using the mean ± standard deviation or number (percentage).

OAB = overactive bladder syndrome, CKD = chronic kidney disease, COPD = chronic obstructive pulmonary disease, CAD = coronary arterial disease, CHF = congestive heart failure, CVA = cerebral vascular disease, OABSS = total score of overactive bladder symptoms scores questionnaire, USS = total scores of urgency severity scales questionnaire, PPBC = patient perception of bladder condition, Pdet.Qmax = detrusor pressure at maximum flow rate.

†Stepwise backward logistic regression analysis was performed using those variables with P < 0.25 at univariate analysis. R^2^ = 0.19.

## References

[b1] Abrams P. . The standardisation of terminology of lower urinary tract function: Report from the Standardisation Sub-committee of the International Continence Society. Neurourol Urodyn 21, 167–178 (2002). 11857671 10.1002/nau.10052

[b2] Chapple C. R. Muscarinic receptor antagonist in the treatment of overactive bladder. Urology 55, 33–50 (2000). 10767450 10.1016/s0090-4295(99)00492-6

[b3] Yiangou Y. . Capsaicin receptor VR1 and ATP-gated ion channel P2X3 in human urinary bladder. BJU Int 87, 774–779 (2001). 11412212 10.1046/j.1464-410x.2001.02190.x

[b4] Apostolidis A. . Decreased sensory receptors P2X3 and TRPV1 in suburothelial nerve fibers following intradetrusor injections of Botulinum toxin for human detrusor overactivity. J Urol 174, 977–982 (2005). 16094018 10.1097/01.ju.0000169481.42259.54

[b5] Kuo H. C. Reduction of urgency severity is associated with long-term therapeutic effect after intravesical onabotulinumtoxin A injection for idiopathic detrusor overactivity. Neurourol Urodyn 30, 1497–1502 (2011). 21717501 10.1002/nau.21132

[b6] Kuo H. C. Urodynamic evidence of effectiveness of botulinum A toxin injection in treatment of detrusor overactivity refractory to anticholinergic agents. Urology 63, 868–872 (2004). 15134967 10.1016/j.urology.2003.12.007

[b7] Sahai A., Khan M. S. & Dasgupta P. Efficacy of botulinum toxin-A for treating idiopathic detrusor overactivity: Results from a single center, randomized, double-blind, placebo controlled trial. J Urol 177, 2231–2236 (2007). 17509328 10.1016/j.juro.2007.01.130

[b8] Brubaker L. . Refractory idiopathic urge urinary incontinence and botulinum A injection. J Urol 180, 217–222 (2008). 18499184 10.1016/j.juro.2008.03.028 PMC2597793

[b9] Anger J. T., Weinberg A., Suttorp M. J., Litwin M. S. & Shekelle P. G. Outcomes of intravesical botulinum toxin for idiopathic overactive bladder symptoms: a systematic review of the literature. J Urol 183, 2258–2264 (2010). 20400142 10.1016/j.juro.2010.02.009 PMC3152380

[b10] Jiang Y. H., Ong H. L. & Kuo H. C. Predictive factors of adverse events after intravesical suburothelial onabotulinumtoxina injections for overactive bladder syndrome-A real-life practice of 290 cases in a single center. Neurourol Urodyn 36, 147–147 (2017). 10.1002/nau.22892 26417884

[b11] Liao C. H. & Kuo H. C. Increased risk of large post-void residual urine and decreased long-term success rate after intravesical onabotulinumtoxinA injection for refractory idiopathic detrusor overactivity. J Urol 189, 1804–1810 (2013). 23178902 10.1016/j.juro.2012.11.089

[b12] Wang C. C., Liao C. H. & Kuo H. C. Diabetes mellitus does not affect the efficacy and safety of intravesical onabotulinumtoxinA injection in patients with refractory detrusor overactivity. Neurourol Urodyn 33, 1235–1239 (2014). 24115083 10.1002/nau.22494

[b13] Kuo H. C., Liao C. H. & Chung S. D. Adverse events of intravesical botulinum toxin a injections for idiopathic detrusor overactivity: risk factors and influence on treatment outcome. Eur Urol 58, 919–926 (2010). 20864251 10.1016/j.eururo.2010.09.007

[b14] Osborn D. J. . Urinary retention rates after intravesical onabotulinumtoxinA injection for idiopathic overactive bladder in clinical practice and predictors of this outcome. Neurourol Urodyn 34, 675–678 (2015). 24975819 10.1002/nau.22642 PMC4755310

[b15] Hsiao S. M., Lin H. H. & Kuo H. C. Factors associated with therapeutic efficacy of intravesical onabotulinumtoxinA injection for overactive bladder syndrome. PLoS One 11, e0147137 (2011). 10.1371/journal.pone.0147137 PMC4732812 26824901

[b16] Chancellor M. B. The overactive bladder progression to underactive bladder hypothesis. Int Urol Nephrol 46 (Suppl 1), S23–27 (2014). 25238891 10.1007/s11255-014-0778-y

[b17] Kuo H. C. Videourodynamic characteristics and lower urinary tract symptoms of female bladder outlet obstruction. Urology 66, 1005–1009 (2005). 16286113 10.1016/j.urology.2005.05.047

[b18] Kuo H. C. Clinical symptoms are not reliable in the diagnosis of lower urinary tract dysfunction in women. J Formos Med Assoc 111, 386–391 (2012). 22817816 10.1016/j.jfma.2011.05.014

[b19] Abrams P. Bladder outlet obstruction index, bladder contractility index, and bladder voiding efficiency: three simple indices to define bladder voiding function. BJU Int 84, 14–15 (1999). 10444116 10.1046/j.1464-410x.1999.00121.x

[b20] Hsiao S. M. . Evaluation of bladder diary parameters based on correlation with the volume at strong desire to void in filling cystometry. PLoS One 8, e69946 (2013). 23922866 10.1371/journal.pone.0069946 PMC3726771

[b21] Nixon A. . A validated patient reported measure of urinary urgency severity in overactive bladder for use in clinical trials. J Urol 174, 604–607 (2005). 16006914 10.1097/01.ju.0000165461.38088.7b

[b22] Homma Y. . Symptom assessment tool for overactive bladder syndrome–overactive bladder symptom score. Urology 68, 318–323 (2006). 16904444 10.1016/j.urology.2006.02.042

[b23] Jiang Y. H., Liu H. T. & Kuo H. C. Decrease of urinary nerve growth factor but not brain-derived neurotrophic factor in patients with interstitial cystitis/bladder pain syndrome treated with hyaluronic acid. PLoS One 9, e91609 (2014). 24614892 10.1371/journal.pone.0091609 PMC3948883

[b24] Altman D. G. Practical statistics for medical research (ed. Altman D. G.) 325–364 (Chapman & Hall, 1991).

[b25] Rosner B. Fundamentals of Biostatistics. (ed. Rosner B.) 577–676 (Duxbury, 2000).

[b26] Rosenbaum P. R. & Rubin D. B. The central role of the propensity score in observational studies for causal effects. Biometrika 70, 41–55 (1983).

[b27] Kaplan E. L. & Myer P. Nonparametric estimation from incomplete observations. J Am Stat Assoc 53, 457–481 (1958).

